# Day care for dementia patients from a family caregiver's point of view: A questionnaire study on expected quality and predictors of utilisation - Part II

**DOI:** 10.1186/1472-6963-11-76

**Published:** 2011-04-13

**Authors:** Carolin Donath, Angelika Winkler, Elmar Graessel, Katharina Luttenberger

**Affiliations:** 1 Medical Psychology and Medical Sociology, Clinic for Psychiatry and Psychotherapy, Erlangen University Hospital, Schwabachanlage 6, 91054 Erlangen, Germany; 2 Alzheimer's Society Brandenburg, Stephensonstraße 24-26, 14482 Potsdam, Germany

## Abstract

**Background:**

The investigation of the predictive variables for utilisation of day care and the views of family caregivers of dementia patients about quality of day care are the goals of this work.

**Methods:**

The cross-sectional study was carried out as an anonymous written survey of family caregivers of dementia patients in Germany. Participants were 404 family caregivers of dementia patients, of these 128 were users of day care, 269 were non-users and 7 gave no details about utilisation. Qualitative and quantitative data were analysed using qualitative content analysis and binary logistic regression analysis.

**Results:**

The assessment of how helpful day care is for the individual care situation and the age of the family caregiver are significant predictors for utilisation of day care. Caregivers most frequently cited a programme of activities suited to the abilities of the dementia patients as quality criterion.

**Conclusions:**

In order to reduce the number of those caregivers who think they don't need day care compared with the number who really don't need it, caregivers should be transparently informed of the relevant advantages and quality principles of using day care. According to caregivers' wishes, the organisation of day care centres must include activities suited for dementia patients.

## Background

In an ever-ageing society, the increase in dementia is becoming a social challenge. Two care problems in particular need to be solved. First, optimisation of the number of patients who receive suitable, effective and desired treatment in order to influence the progression of the illness as positively as possible [1,2], and second, optimisation of the support for family caregivers.

Exactly how caregivers can be supported must also be researched. This is primarily a search for effective methods of support [3-5], but more importantly, it should answer the question about how to motivate family caregivers and also convince them to avail themselves of support services.

Although there are studies about the effectiveness of day care [5], the question of which factors influence the utilisation of day care has remained mostly unanswered in publications to date. A systematic search of three databanks - Medline^®^, PsychInfo^® ^and Cinahl^® ^shows that there has been little scientific research in this area to date. There are very few empirical studies about utilisation, all of them carried out in the U.S., and only two studies about quality. Specific studies regarding day care for dementia patients alone are scarce, since several respite offers were considered jointly in the published surveys.

In day care, the patient is cared for with other dementia patients for up to eight hours a day by professionals, trained nurses in particular, in an out-patient facility. In some instances, non-pharmacological therapies to promote cognitive and everyday practical skills are offered. Normally the patient is collected by a transport service and brought home again at the end of the day [6-8]. Day care relieves the family caregiver directly of nursing duties and has proved itself in a meta-analytical study as effective in reducing caregivers' subjective burden and depression [5]. The degree of usage of day care varies from 4.2% to 61.0% [9-12]. In order to specifically influence this low rate - in the whole of Europe only 4.3% - it is important to know the predictors for utilisation and particularly those that represent the perspective of the family caregivers.

Andersen's model of utilisation of health care services [13] revealed that the factors which influence the usage of health services can be arranged in the categories "predisposing", "enabling" and "need" variables. Toseland and his colleagues [14] maintain that predisposing factors and enabling factors are more important than need factors in predicting usage of services. But they also report that there are studies in which the "need" variables were the most important predictors for usage of health services. Our study is intended to investigate which variables of these three predictor groups are really important for the use of day care, especially since the importance of the three factors differs depending on the type of service.

Fulfilment of quality standards is the main characteristic of a professional service. These quality standards normally reflect expert opinion. The quality of day care, particularly its "user-friendliness," would be improved if the "customer's" - i.e. family caregiver's - concept of quality were systematically taken into consideration. To date, no scientific study on quality, which includes the quality wishes of dementia caregivers exclusively for day care, has been published.

The *first objective *of the study constitutes: Which variables concerning family caregiver's and patient's characteristics influence the utilisation of day care?

The *second objective *is: Which quality characteristics of day care from a family caregiver's point of view, should be fulfilled, dependent on whether day care has already been used ("user") or not ("non-user")?

## Methods

### Design

The data basis of the study is a written anonymous survey of family caregivers of dementia patients living in the community. The cross-sectional study was carried out in four regions in Germany - in Erlangen and district (Southern region), in Dortmund and district (West), in Kassel and district (North central) and in the Federal State of Brandenburg, specifically in the region around Potsdam (Northeast). Each study region had urban and rural areas with a minimum number of 250,000 inhabitants and therefore at least 2,500 dementia patients.

The survey papers consisted of a letter, the questionnaire, a stamped addressed envelope and an information brochure titled "Das Wichtigste über die Alzheimer-Krankheit" (The most important facts about Alzheimer's disease) [15]. The anonymity of the study in particular was emphasised in the letter. The objective was, on the one hand to reach family caregivers who had no previous experience with day care ("non-users") and on the other to gain information from those who had already used or were using it ("users").

200 questionnaires were to be distributed in each study region within the recruitment period of six months through the Medical Services of the Health Insurance offices at the initial appraisal within the framework of the long-term nursing care regulations. This procedure initiates access to the financial resources which allow the caregivers to take up offers of respite care. Hence this recruiting pathway facilitated the contact to dementia caregivers, who in most cases had never used day care. If one of the two care diagnoses made at the initial appraisal was "dementia," the survey papers were to be given to the family caregiver.

300 questionnaires in each study region were to be distributed through the regional offices of the Alzheimer's Society and other caregiver counselling services. Using this recruiting pathway, there is a good chance that a family who was already using one service (in our study "caregiver counselling") might also have had some experience with other relief offers such as day care [11].

Of the 2,000 questionnaires (500 in each region) which were sent to the distributors, 404 were returned, giving an inter-regional response of at least 20.2%.

### Instruments

The 3-page questionnaire was tested on 12 family caregivers for comprehensibility and acceptability in a pilot phase.

Quantitative data [in brackets the Andersen-model category of the variable]: Besides the socio-demographic variables (see Table 1) [predisposing], characteristics of the care situation and variables in connection with day care were collected. The care situation is fundamentally characterised by the amount of care time required by the caregiver [need], whether he/she gets help from others [need] and whether the patient has been classified within the health care insurance system [enabling]. First, the "day care" offer was described briefly. Then the questions "Do you know about day care?" [enabling] and "Do you use day care?" [predisposing] were to be answered dichotomously (yes/no). The assessment of how helpful day care would be to the family caregiver in his/her care situation was carried out on a 5-step self-report scale (from "0 = I don't need it" to "4 = I need it urgently") [need] with the addition of "independent of whether you have previously used day care or not". In addition, family caregivers were to grade the accessibility of day care facilities according to the three categories "Don't know", "Not easily accessible" or "Accessible" [enabling] and whether they were living in a rural or urban area [enabling]. Therefore variables from all three categories of the Andersen model were assessed.

**Table 1:** Sample characteristics of family caregivers and dementia patients

Variable	Mean (SD)	Frequency (%)
Family caregiver		
Age (years)	61.3 (11.9)	
Gender (female)		73.3
Education level		
No school leaving certificate		0.8
Secondary school (9 years)		47.7
Vocational school (10 years)		30.7
Grammar school (13 years)		20.8
Employed		28.6
Relationship to patient		
Spouse		43.8
Adult children		48.9
Others		7.3
Place of residence (city)^a^		44.4
Caregiver/patient sharing accommodation (apartment/house)		75.0
No caregiving help by others		34.8
Dementia patient		
Age (years)	78.8 (9.1)	
Gender (female)		63.6
Level of care (health insurance)^b^		
Not yet applied for		5.8
Applied for		19.2
Level 1		29.6
Level 2		31.6
Level 3		13.7
Duration of dementia (years)	4.2 (3.3)	
Time for care per day (hours)	5.1 (4.7)	

^a ^100,000 or more inhabitants ( = city)

^b ^the higher the level the higher the necessary help

Qualitative data: The data for qualitative analysis was collected using an open question: "Independent of whether you have already used day care or not: What would you personally expect from a "good" day care facility?" The collection of data on the quality was carried out using open questions for two reasons: (a) A standardised, validated questionnaire that included all relevant quality aspects did not exist. (b) The "free recall" of one's own reflections represents the family caregiver's concept of quality without being influenced by extrinsic arguments.

### Description of the sample

The features of the family caregivers and their dementia patients can be seen in Table 1.

### Ethical Considerations

This is a cross-sectional written questionnaire study. The ethically relevant aspects of "voluntary disclosure of information" and "anonymity of answers" were dealt with in the accompanying letter, which was accepted by the Board of the regional Alzheimer's Society and presented to the Management of the German Alzheimer's Society for approval. The University Erlangen-Nuremberg ethics committee reviewed the study design and waived the need for ethical approval. The ethical board of the Alzheimer's Society also declared that a formal approval was not necessary.

### Statistical procedure

Quantitative data analysis: The quantitative questions were answered by 97.4% of the 404 family caregivers on average. In order to ascertain which variables significantly influence the utilisation of day care, binary logistic regression analysis was carried out. The dichotomous dependent variable was the "use" (code = 1) or "non-use" (0) of day care. The coding of potential predictors is shown in the legend to Table 2. A multicollinearity test was carried out before the regression analysis in order to exclude confounded variables because of a significant correlation of moderate strength (r > 0.40). Therefore the following variables were not included in the multivariate regression analysis: relationship between caregiver and dementia patient, shared accommodation, employed caregivers and level of care (health insurance). The explained variance in the regression model is quoted with Nagelkerke's R². The significance of potential predictors was measured using Wald's coefficient (α = 0.05).

**Table 2:** Binary logistic regression analysis with use of day care as dependent variable (N = 404)

	Regression coefficient β	P	Odds ratio	95% Confidence interval for odds ratio	
				Lower value	Higher value
Gender of family caregiver ^a^	0.17	0.70	1.19	0.49	2.91
**Age of family caregiver**	0.04	**0.05**	1.04	1.00	1.07
School leaving certificate (1)^b^	0.24	0.62	1.27	0.50	3.25
School leaving certificate (2)^b^	0.06	0.90	1.06	0.42	2.67
Gender of dementia patient^1^	-0.06	0.90	0.95	0.40	2.26
Age of dementia patient	0.00	0.85	1.00	0.96	1.05
Help from others (informal or formal caregivers)^c^	-0.45	0.25	0.64	0.30	1.37
Duration of illness	< 0.001	0.97	1.00	0.99	1.01
Hours per day spent on care	-0.04	0.37	0.96	0.88	1.05
Place of residence (city)^c^	-0.10	0.78	0.91	0.45	1.83
"Knowing" about day care ^c^	-20.21	1.00	< 0.001	< 0.001	.
**"Need" for day care**	1.07	**<.001**	2.92	2.19	3.89
Accessibility of day care facilities (1)^d^	-0.75	0.07	0.47	0.21	1.07
Accessibility of day care facilities (2)^d^	-1.39	0.06	0.25	0.06	1.08

^a^Gender: 0 = male, 1 = female

^b ^School leaving certificate: 1 = secondary school, 2 = vocational school, reference value: grammar school

^c ^0 = yes 1 = no

^d ^Accessibility of day care facilities: 1 = don't know, 2 = not easily accessible, reference value: accessible

df: number of degrees of freedom

p: significance

Qualitative data analysis: The question about the quality of day care was evaluated using Morgan's [16] content analysis method. Two researchers undertook assignment to the categories independent of each other. In individual cases of divergence, consensus had to be achieved. The frequency of citation of the categories is shown in Table 3. The design respects general performance criteria of qualitative research as described by Mayring [17] such as that the research process should follow clear rules, be exactly documented and that interpretation be well-founded.

**Table 3:** Family caregivers' quality requirements (N = 269) - ranking of most frequently cited requirements (≥ 5%)^a^

Users^b^: Quality requirement (classification^d^)	Number (%)	Non-users^c^: Quality requirement (classification^d^)	Number (%)
Activities (as regards content): general type, exercise, games, etc (P I)	33 (31)	Activities (as regards content): general type, exercise, games, etc (P I)	30 (19)
Activities (formal): regarding abilities, varied, sensible (P II)	21 (19)	Affectionate manner (incl. empathy) (P II)	19 (12)
Affectionate manner (incl. empathy) (P II)	14 (13)	Qualified/well-trained staff (S II)	18 (11)
Good manner (P II)	13 (12)	Dementia patient should feel at ease (E I)	17 (11)
Qualified/well-trained staff (S II)	12 (11)	Reliable staff (P II)	12 ( 7)
Friendly staff (P II)	10 ( 9)	Existing abilities maintained (E I)	11 ( 7)
Existing abilities maintained (E I)	9 ( 8)	Organised transport (S I)	10 ( 6)
Promote social contact (E I)	9 ( 8)	Good manner (P II)	10 ( 6)
Dementia patient should feel at ease (E I)	9 ( 8)	Friendly staff (P II)	9 ( 6)
Considerate of dementia patients' needs (P II)	7 ( 7)	Considerate of dementia patients' needs (P II)	8 ( 5)
Reliable staff (P II)	6 ( 6)	Respite for family caregivers (E II)	8 ( 5)
Small groups (S I)	6 ( 6)		

^a ^multiple answers were possible

^b ^128 family caregivers (31.7%) were users; of these 108 supplied details of quality requirements (࣓ 100%)

^c ^269 family caregivers (66.6%) were non-users; of these 161 supplied details of quality requirements (࣓ 100%) 7 family caregivers (1.7%) gave no details about utilisation

^d ^Classification of quality criteria:

• Structural quality (S):

I. Non-personal factors (S I)

II. Person-related factors (S II)

• Process quality (P):

I. Content aspects of procedure (What is done?) (P I)

II. Formal aspects of procedure (How is it done?) (P II)

• Quality of result (E):

I. Aims concerning dementia patients (E I)

II. Aims concerning family caregivers (E II)

Finally, the individual conclusions were assigned to the three quality categories, "Structure", "Process" and "Quality of results" by two researchers independent of each other.

## Results

### Quantitative Analysis

At 77.6%, the existence of day care facilities is well known to family caregivers. About one-third of the respondents (32.2%) use them. In the assessment of how helpful this offer is, two-fifths of the responding family caregivers need day care very urgently (20.1%) or urgently (21.5%) while almost the same number think they don't need it at all (27.4%) or hardly ever (15.0%). The remaining 16.0% were indifferent ("might need it to some extent").

More than half of the family caregivers stated that there was an accessible day care facility in their locality (56.1%). One-third (35.8%) knew nothing about the distance to or the accessibility of their nearest day care facility, while 8.1% of the respondents said that day care facilities were not easily accessible for them.

Binary logistic regression analysis showed that with an explained variance of 60.1% (R²) a significant regression model (χ^2 ^(15) = 169.549; p < 0.001), characterised by two significant predictors (Table 2) could be identified. So 84.5% of the respondents could be correctly assigned to the categories "user" or "non-user". The probability of using day care increases almost threefold when the degree of "need" increases (p < 0.001).

As the family caregivers get older, the chances of their utilising day care are also significantly greater (p = 0.05). The probability of utilisation tends to be related to the estimated accessibility of day care facilities. It decreases when the nearest facility is unknown (p = 0.07) or thought to be difficult to access (p = 0.06) compared to the grading "is accessible for me". Utilisation is not associated with the age of the dementia patient, gender of either the family caregiver or dementia patient, level of education, rural versus urban living, help from others, hours per day spent on care or with the duration of the patient's illness.

### Qualitative Analysis

The family caregivers' top three quality requirements for day care partially overlap for users and non-users. Top of the list were useful activities such as exercise and games (31% and 19%, Table 3). In second place, their wishes vary. While 19% of the users cite the formal aspect of an activity suitable for the patients' abilities, 12% of the non-users expect the staff to treat the dementia patient in an affectionate manner. This aspect of quality was cited in third place (13%) by the users, while the non-users cited well-trained staff (11%) and a day care center where dementia patients feel at ease. For the users, the content and the formal aspects of the activity programme are by far the most important (50%) followed by their wishes about the manner of dealing with dementia patients (affectionate, friendly, good: 34%).

## Discussion

Although the objectives of this study, utilisation and quality, are highly relevant for the further development of the content of respite offers, there has been little scientific research in these areas to date. Therefore, scientifically we are breaking new ground with this study.

The sample of family caregivers was taken in four regions, throughout Germany, which were suitable from an urban and rural point of view with varying coverage of day care facilities. It was not, however, a representative sampling. The response rate was more than 20%. This value corresponds exactly with the empirically-established response in anonymously written surveys of family caregivers who received no reward for answering [18,19]. However, a recruitment bias cannot be ruled out. The frequencies of the citation of the individual quality criteria should therefore be regarded as an initial orientation. But by using various recruiting pathways, it was possible to affirm that a substantial fraction of the family caregivers had never used day care previously, which would also be expected in a representative sample. The fact that over two-thirds were non-users has also been seen in other studies, where utilisation rates of 4% and 15% respectively are reported [9,11]. It is not justified by the study to generalise on the Health Care System of other countries. The data were only collected from caregivers living in Germany and thus living under the legislation of the "Social Care Insurance" which allows caregivers to get costs which relate to the usage of caregiver support respectively respite services refunded.

In this study, we were able to establish that day care is very well known (awareness level 78%). The awareness of day care in international studies fluctuates between 33% and 94% [11,12,20,21]. Hence the first pre-requirement for utilisation is fulfilled. Brodaty et al. [22] however report that the utilisation of respite offers by family caregivers is low, even though these are well known and even when the services are free-of-charge [10]. This means that there must be other more significant variables that determine utilisation. In our study, the family caregivers' assessment of the helpfulness of day care centres ("I need them") is a highly significant predictor for using it. This corresponds with the findings of Gill, Hinrichsen and DiGiuseppe [[23] for in-home and out-of-home services], who showed that need variables explain more variance in service utilisation than predisposing or enabling variables speaking in terms of the Andersen model.

In a study by Toseland et al. [14], it was demonstrated that this assessment of helpfulness is not a significant predictor for utilisation in the range of professional health and human services. It must be taken into consideration that this study is not specific to any one offer, either. Utilisation which depends on how well known the service is, is significantly moderated by the assessment of helpfulness when the utilisation refers to a single service, as in our study about day care. Here 42% said that they needed day care urgently or very urgently. This percentage is markedly higher than Toseland's et al. [11] (10%) or that of another study [12]. Van Exel et al. report that 19% of their interviewees say that they need day care. In accord with the results of the regression analysis in our study, Kosloski and Montgomery [10] stated that estimating the need as in accepting the helpfulness of an offer is a significant predictor for utilisation. If there are plans to relieve as many family caregivers as possible, the service should not only be made known to family caregivers but the individual advantages of utilising day care should also be pointed out.

In our study, utilisation of day care was positively associated with the age of the family caregiver. This was also seen in the study by van Exel et al. [12] but not in those by Douglass and Visconti [24] or Montoro-Rodriguez et al. [25]. Concurring with the aforementioned authors, it could be shown that neither the gender or level of education of the family caregiver, the number of hours per day spent on care nor residence in urban or rural areas has any influence on the utilisation of day care.

All in all, only 8% of respondents maintained that day care facilities were difficult to access for them. This value is comparable with those in other studies (9%) [11]. Lack of knowledge about the nearest day care facility is more predominant at 36%. The same value was observed by Toseland et al. [11]. Based on this high percentage and the findings in other studies that easy access to respite offers is positively associated with utilisation [10,14,25] - a tendency that was also noted in our study - one practical objective should be to explain the helpfulness of this offer to the family caregiver and to give clear information about the location of the nearest day care facility and directions how to get there. Other authors have shown that usage of day care predicts institutionalisation. Gaugler et al. [26] showed generally a protective effect for day care users concerning institutionalisation. McCann and colleagues [27] showed that the protective effect seems to work especially for males. Besides the cost-effectiveness effects there are positive effects of usage of day care on caregivers' health: Although in reviews [28] and in meta-analytical studies [29] the moderately positive effects of respite on subjective burden and depression of family caregivers have now been proven, little is known about what an effective intervention should be like.

The participants in this study, family caregivers, both users and non-users all most frequently cited the quality criterion of an activity programme, suitable for the individual abilities of dementia patients. They place particular value on process quality in the form of an "affectionate", "friendly" manner of dealing with dementia patients.

The importance of an empathetic respectful manner as a quality criterion is found in other studies, although they were not exclusively about day care. In a study by Winslow [30], in which 21 dementia family caregivers were asked in an open question about the quality of various community services on offer, including day care, the utilisation of day care was in second place. Sormunen et al. [31] observed quality aspects using Dementia Care Mapping of 85 dementia patients in either residential or day care units.

As no empirical studies about the quality requirements of family caregivers of dementia patients regarding day care in particular have been published to date, we would like to include via this study the family caregivers' views in the scientific discussion about quality standards of dementia-specific day care. In research into community services for patients who are cared for at home, more consideration of the unmet needs of family caregivers is called for [21]. This is very important, because consideration of the family caregivers' quality requirements reduces the barrier to utilisation [30].

## Conclusions

In order to increase the utilisation of day care to relieve family caregivers, and thus to reduce the number of those caregivers who think they don't need day care compared with the number who really don't need it, the caregivers should not only be transparently informed about the existence of this offer but also about the advantages for themselves.

Information about the location and accessibility of the nearest day care facility should be provided to all family caregivers. Dementia-specific day care programmes should have a programme of activities geared to the individual abilities of dementia patients. In order to improve the acceptance of respite care by family caregivers, it is sensible to allow them to spend some time observing to convince them of the friendly and respectful manner in which the patients are treated.

## Competing interests

The authors declare that they have no competing interests; especially do the authors not have any conflict of interest towards organisations offering day care.

## Authors' contributions

EG designed the study and its questionnaires, supervised data collection, was responsible for the interpretation of data and took part in writing the paper. AW assisted with designing the study, organised the data collection and gave important hints for interpretation and discussion. CD designed the study, was responsible for the statistical design and data analysis and took part in writing the paper. KL participated in its design and statistical analysis, took part in the interpretation and helped to draft the manuscript. All authors read and approved the final manuscript.

## Pre-publication history

The pre-publication history for this paper can be accessed here:

http://www.biomedcentral.com/1472-6963/11/76/prepub
